# Optimisation of Ultrasound-Assisted Extraction Conditions for Phenolic Content and Antioxidant Capacity from *Euphorbia tirucalli* Using Response Surface Methodology

**DOI:** 10.3390/antiox3030604

**Published:** 2014-09-17

**Authors:** Quan V. Vuong, Chloe D. Goldsmith, Trung Thanh Dang, Van Tang Nguyen, Deep Jyoti Bhuyan, Elham Sadeqzadeh, Christopher J. Scarlett, Michael C. Bowyer

**Affiliations:** 1 Pancreatic Cancer Research, Nutrition Food & Health Research Group, School of Environmental and Life Sciences, University of Newcastle, NSW 2258, Australia; E-Mails: chloe.d.goldsmith@uon.edu.au (C.D.G.); trung.dang@uon.edu.au (T.T.D.); vantang.nguyen@uon.edu.au (V.T.N.); deepjyoti.bhuyan@uon.edu.au (D.J.B.); elham.sadeqzadeh@newcastle.edu.au (E.S.); c.scarlett@newcastle.edu.au (C.J.S.); michael.bowyer@newcastle.edu.au (M.C.B.); 2 Faculty of Food Technology, Nha Trang University, No. 2 Nguyen Dinh Chieu, Nha Trang, Khanh Hoa 8458, Vietnam

**Keywords:** antioxidant, *Euphorbia tirucalli*, ultrasonic-assisted extraction, optimization, phenolic compounds, response surface methodology

## Abstract

*Euphorbia tirucalli* (*E. tirucalli*) is now widely distributed around the world and is well known as a source of traditional medicine in many countries. This study aimed to utilise response surface methodology (RSM) to optimise ultrasonic-assisted extraction (UAE) conditions for total phenolic compounds (TPC) and antioxidant capacity from *E. tirucalli* leaf. The results showed that ultrasonic temperature, time and power effected TPC and antioxidant capacity; however, the effects varied. Ultrasonic power had the strongest influence on TPC; whereas ultrasonic temperature had the greatest impact on antioxidant capacity. Ultrasonic time had the least impact on both TPC and antioxidant capacity. The optimum UAE conditions were determined to be 50 °C, 90 min. and 200 W. Under these conditions, the *E.* *tirucalli* leaf extract yielded 2.93 mg GAE/g FW of TPC and exhibited potent antioxidant capacity. These conditions can be utilised for further isolation and purification of phenolic compounds from *E. tirucalli* leaf.

## 1. Introduction

*Euphorbia tirucalli* (*E.* *tirucalli*) is native to Madagascar and Africa, but it is now widespread around the world because of its tolerance to a wide range of climatic conditions [1,2]. *E.* *tirucalli* has been used as folk medicines in the Middle East, India, Africa and South America for the treatment of a range of ailments including syphilis, asthma, cancer, colic, intestinal parasites and leprosy [3,4,5]. Recently, it has been linked to other benefits including hepatoprotective, antimicrobial, antioxidant, insecticidal, larvicidal, molluscicide and antiarthritic activities [6], which have resulted in significant scientific interest in the phytochemical profile of the plant [1].

Phenolic and terpenoid compounds have been identified in the phytochemical profile of *E.* *tirucalli*, and extracts have been shown to possess potent antioxidant properties [7,8]. Phenolic compounds are major bioactive compounds in medicinal plants and have been reported as powerful antioxidants and health promoters. The phenolic profile and antioxidant properties of *E.* *tirucalli* have been reported in a previous study, which found that acetone extracts yielded a higher concentration of phenolics than more polar solvent systems (80% aqueous methanol) [8]. However, optimal extraction conditions were not investigated and further study is required to fully characterise the phenolic profile.

Ultrasonic-assisted extraction (UAE) has been shown to be a fast and effective technique for extracting phytochemicals from plant materials that is easily up-scaleable [9]. Ultrasonic parameters such as temperature, time and power have been reported to exert significant impact on the extraction yield of plant phytochemicals and antioxidants [10,11,12]. A mixture of ethyl acetate: ethanol (4:1 v/v) was reported to efficiently extract triterpenoids from the root of *Euphorbia pekinensis* Rupr [13]. Therefore, this solvent mixture was used in this study and it was hypothesised that UAE temperature, time and power had significant effects on extraction efficiency of total phenolic compounds (TPC) and total antioxidant capacity (TAC) of the extracts from *E.* *tirucalli* leaf and the optimal extraction conditions could be established using response surface methodology (RSM).

The current study aimed to optimise the UAE parameters of temperature, time and power for the extraction of phenolics from *E.* *tirucalli*. These optimal conditions can be applied for further isolation and purification of phenolic compounds from *E.* *tirucalli*.

## 2. Experimental Section

### 2.1. Plant Materials

The leaf of the *Euphorbia tirucalli* tree (phylloclades) was collected on July 16, 2013 from a property located in Saratoga, NSW, Australia (33.47° S, 151.35° E). The leaf was then immediately transferred to the laboratory and stored at −20 °C to minimise phenolic degradation. Before commencing experiments, the leaf was immersed in liquid nitrogen, then particulated using a commercial blender. The fresh ground leaf was then stored at −20 °C until required.

### 2.2. Ultrasound-Assisted Extraction (UAE)

A mixture of ethyl acetate: ethanol 4:1 (v/v) was utilized as the solvent system for optimisation of UAE conditions and was applied at a solvent-to-sample ratio of 100:32 mL/g of fresh leaf weight. The ground *E.* *tirucalli* leaf was placed in the extraction chamber, and filled with extraction solvent. The extraction chamber was completely immersed into an ultrasonic bath (Soniclean, 220 V, 50 Hz and 250 W, Soniclean Pty Ltd., Thebarton, Australia) with pre-set conditions for temperature, time and power as designed by response surface methodology software. When the ultrasonic extraction was completed, the extracts were immediately cooled on ice to room temperature, filtered using a 5 mL syringe fitted with a 0.45 μm cellulose syringe filter (Phenomenex Australia Pty. Ltd., Lane Cove, Australia) and diluted to the required volume for quantitative analysis.

### 2.3. Response Surface Methodology (RSM)

A response surface methodology (RSM) approach with a Box-Behnken design was employed to design experimental conditions to investigate the influence of the three independent ultrasonic parameters: temperature (30, 45, and 60 °C), time (30, 45, and 60 min) and power (60%, 80%, and 100% or 150, 200, and 250 W).

The independent variables and their code variable levels are shown in Table 1. To express the level of total phenolic compounds (TPC) and total antioxidant capacity (TAC) as a function of the independent variables, a second-order polynomial equation was used as follows [14]:

(1) $$Y=\beta_{o}+\sum_{i=1}^{k}\beta_{i}{Χ}_{i}+\sum_{\begin{array}{l} i=1\\ i<j \end{array}}^{k-1}\sum_{j=2}^{k}\beta_{ij}{Χ}_{i}{Χ}_{j}+\sum_{i=1}^{k}\beta_{ii}{Χ}_{i}^{2}$$

where various *X_i_* values are independent variables affecting the responses *Y*; *β*_0_, *β_i_*, *β_ii_* *and β_ij_* are the regression coefficients for intercept, linear, quadratic, and interaction terms, respectively; and *k* is the number of variables.

The three independent ultrasonic parameters were assigned as; *X*_1_ (temperature, °C), *X*_2_ (time, min.) and *X*_3_ (power, %). Thus, the function containing these three independent variables is expressed as follows:

(2) $$Y=\;\beta_{0}+\beta_{1}X_{1}+\beta_{2}X_{2}+\beta_{3}X_{3}+\beta_{12}X_{1}X_{2}+\beta_{13}X_{1}X_{3}+\beta_{23}X_{2}X_{3}+\beta_{11}X_{1}^{2}+\beta_{22}X_{2}^{2}+\beta_{33}X_{3\;}^{2}$$

### 2.4. Determination of Total Phenolic Content (TPC)

The extract was diluted 20× to fit within the optimal absorbance range for colorimetric assessment of total phenolic compounds (TPC), which was determined according to the method of Vuong *et al*. [15]. Gallic acid was used as the standard for the construction of a calibration curve, with the results expressed as mg of gallic acid equivalents per gram of fresh weight (FW) (mg GAE/g).

**Table 1:** Box-Behnken design and observed responses

Run	Ultrasonic Conditions			Experimental Values (*n* = 3)				
	Temperature (°C)	Time (min)	Power (%) *	TPC (mg GAE/g)	Antioxidant Capacity			
					ABTS (%)		DPPH (%)	CUPRAC (mM TE/g)
**1**	30	30	80	2.99	42.81	17.80		37.07
**2**	30	60	60	2.05	56.31	21.94		42.74
**3**	30	60	100	2.40	50.20	19.22		41.01
**4**	30	90	80	2.34	52.59	18.12		36.61
**5**	45	30	60	2.20	74.88	25.17		56.66
**6**	45	30	100	2.99	74.01	27.32		67.63
**7**	45	60	80	2.71	66.07	30.25		55.67
**8**	45	60	80	3.11	46.91	18.58		38.56
**9**	45	60	80	3.51	74.52	25.17		66.72
**10**	45	90	60	2.63	64.23	23.43		46.81
**11**	45	90	100	3.34	74.27	30.28		67.00
**12**	60	30	80	2.05	74.52	20.90		71.70
**13**	60	60	60	2.39	53.82	47.46		51.95
**14**	60	60	100	2.83	69.13	43.27		57.17
**15**	60	90	80	3.12	62.22	39.66		48.13

* 60%, 80% and 100% power were equivalent to 150, 200 and 250 W.

### 2.5. Determination of Antioxidant Capacity

*ABTS total antioxidant capacity*: The extract was diluted 40× to fit within the optimal absorbance range for colorimetric assessment. Total antioxidant capacity (TAC) was measured using 2,2′-azino-bis-3-ethylbenzothiazoline-6-sulphonic acid (ABTS) assay as described by Thaipong *et al*. [16]. Results were expressed as percentage of inhibition and were calculated using the formula:

TAC (%) = (*Abs_control_* – *Abs_sample_*) × 100/*Abs_control_* (3)

where *Abs_control_* = control absorbance and *Abs_sample_* = sample absorbance.

*Free radical scavenging capacity:* The extract was diluted 40× and analyzed using 1,1-diphenyl-2-picrylhydrazyl (DPPH) assay as described by Vuong *et al*. [15] with results expressed as percentage of inhibition, calculated according to Equation 3.

*Cupric reducing antioxidant capacity (CUPRAC)*: The extract was diluted 40× and its iron chelating capacity analyzed using cupric ion reducing antioxidant capacity (CUPRAC) assay as described by Apak *et al*. [17]. Trolox (6-hydroxy-2,5,7,8-tetramethylchroman-2-carboxylic acid) was used as the calibration standard, with results expressed as mM of trolox equivalents per g of fresh weight (mM TE/g).

### 2.6. Statistical Analysis

RSM experimental design and analysis were conducted using JMP software (Version 11, SAS, Cary, NC, USA). The software was also used to establish the model equation to graph the 3D- and 2D-contour plots of variable responses, and to predict optimum values for the three response variables. The Student’s *T*-test (conducted using the SPSS statistical software version 20, IBM, Armonk, NY, USA) was used for comparison of the means analysis. Differences between the mean levels in the different experiments were taken to be statistically significant at *p* < 0.05.

## 3. Results and Discussion

### 3.1. Fitting of the Models for Prediction of Total Phenolic Content and Total Antioxidant Capacity

**Figure 1:**
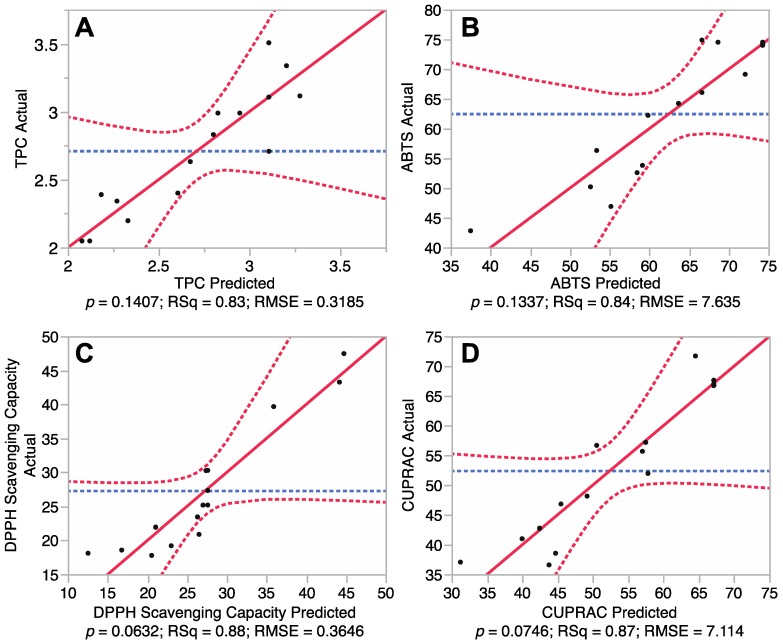
Correlations between predicted and experimental total phenolic content and antioxidant capacity. (**A**) Phenolic content; (**B**) 2,2′-azino-bis-3-ethylbenzothiazoline-6-sulphonic acid (ABTS) antioxidant capacity; (**C**) 1,1-diphenyl-2-picrylhydrazyl (DPPH) free radical scavenging capacity; and (**D**) Cupric reducing antioxidant power (CUPRAC).

It is necessary to test the reliability of the RSM mathematical model in predicting optimal variances and accurately representing the real interrelationships between the selected parameters. Therefore, fitting of the models for total phenolic content and antioxidant capacity of the euphorbia extracts was undertaken. Results of analysis of variances of the Box-Behnken design are shown in Figure 1 and Table 2. Figure 1 revealed a correlation between the predicted and experimental values, while Table 2 presents the summary of variance analysis.

**Table 2:** Analysis of variance for determination of model fitting.

Sources of Variation	TPC	Antioxidant Capacity		
		ABTS	DPPH	CUPRAC
**Lack of fit**	0.77	0.0007 *	0.13	0.0025 *
***R*^2^**	0.83	0.84	0.88	0.87
**Adjusted *R*^2^**	0.53	0.54	0.67	0.65
**PRESS**	3.73	4661.69	2121.07	4042.87
**F Ratio of Model**	2.73	2.81	4.23	3.88
***p* of Model > F**	0.14	0.13	0.06	0.07

* Significantly difference with *p* < 0.05.

Figure 1A showed that the model TPC outputs did not differ significantly from the experimental values (*p* > 0.05). Furthermore, the coefficient of determination (*R*^2^) of the model was 0.83, indicating that 83% of the experimental data can be predictively matched against the model data for TPC. Table 2 also showed that the *p* value for “lack of fit” was 0.77, indicating that the lack of fit was not significant (*p* > 0.05). In addition, the Predicted Residual Sum of Square (PRESS) for the model (a measure of how well the predictive model fits each point in the design) [12], was 3.73 and the F value of the model was 2.73, further revealing that the mathematical model was adequate for prediction of TPC and was fitted to the following second-order polynomial formula:

(4) $$Y_{TPC}=\;3.1067\;+0.0754X_{1}+0.1503X_{2}+0.2863X_{3}+0.4284X_{1}X_{2}+0.0224X_{1}X_{3}-0.0157X_{2}X_{3}-0.4273X_{1}^{2}-0.0556X_{2}^{2}-0.2621X_{3}^{2}\;$$

Fitting of the models for three different antioxidant properties including total antioxidant capacity (ABTS), DPPH free radical scavenging capacity and cupric reducing antioxidant capacity (CUPRAC) were also tested. The results (Figure 1B–D) revealed *p* values for ABTS, DPPH and CUPRAC of 0.13, 0.06 and 0.07, respectively, indicating that there was no significant difference between the predicted values and experimental values (*p* > 0.05). Coefficient of determination (*R*^2^) for the ABTS, DPPH and CUPRAC models (Table 2) were determined to be 0.84, 0.88 and 0.87, respectively, revealing a close correlation between the predicted values and experimental values, with at least 84% of data matching. PRESS values (4661, 2121 and 4042) and *F* values (2.81, 4.23 and 3.88) revealed that these mathematical models were reliable predictors of antioxidant capacity and could be fitted to the following second-order polynomial formulas:

(5) $$Y_{ABTS}=\;74.27\;+7.22X_{1}+2.13X_{2}+3.59X_{3}-0.42X_{1}X_{2}-3.95X_{1}X_{3}-2.13X_{2}X_{3}-10.94X_{1}^{2}-0.19X_{2}^{2}-11.06X_{3}^{2}$$

(6) $$Y_{ABTS}=\;74.27\;+7.22X_{1}+2.13X_{2}+3.59X_{3}-0.42X_{1}X_{2}-3.95X_{1}X_{3}-2.13X_{2}X_{3}-10.94X_{1}^{2}-0.19X_{2}^{2}-11.06X_{3}^{2}$$

(7) $$Y_{ABTS}=\;74.27\;+7.22X_{1}+2.13X_{2}+3.59X_{3}-0.42X_{1}X_{2}-3.95X_{1}X_{3}-2.13X_{2}X_{3}-10.94X_{1}^{2}-0.19X_{2}^{2}-11.06X_{3}^{2}$$

### 3.2. Effects of Ultrasonic Variables on Total Phenolic Content of E. tirucalli Extracts

**Figure 2:**
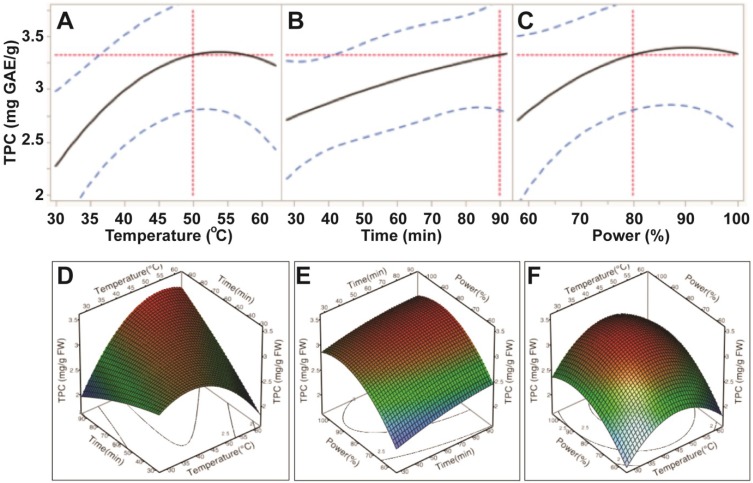
Impact of ultrasonic temperature (30–50 °C), time (30–90 min) and power (60%–100% or 150–250 W) on total phenolic compounds. The 2D impact of temperature, time and power were expressed in Figure 2A–C; while their 3D effects were shown in Figure 2D–F.

Our data showed that the extraction efficiency of TPC had a positive correlation to the three experimental variables; bath temperature (30–50 °C), sonication time (30–90 min.) and sonication power (60%–80%; 150–200 W). Levels of TPC increased steadily when ultrasonic temperature increased from 30 °C to 50 °C; however, the levels of TPC decreased when temperature exceeded 55 °C (Figure 2). Levels of TPC also increased when ultrasonic time increased to 90 min. However, both ultrasonic temperature and time did not significantly affect levels of TPC (*p* > 0.05; Table 3), indicating that these two factors had the least impact on extraction efficiency of TPC. This demonstrated that ultrasonic power was the only parameter to significantly influence the extraction efficiency of TPC from *E.* *tirucalli* leaf (*p* < 0.05; Table 3). Levels of TPC plateaued when ultrasonic power exceeded 200 W (80%) (Figure 2). Therefore, the maximum TPC could be obtained at ultrasonic-assisted extraction conditions of 50 °C, 90 min and 200 W.

The impact of ultrasonic parameters on profile of the TPC extraction curve was similar to findings in previous studies examining seed cake extracts [10], olive pomace [12] and procyanidins removal from *Larix gmelinii* bark [18], which reported that the general application of higher temperatures, longer extraction times and/or higher sonication power increased TPC yields. The rising profile may be explained by two distinct processes; namely, a fast extraction of phenolics from cells close to the surface of the plant material, which are quickly solubilised, and slower diffusion and osmosis-based processes (known as “slow extraction”) involving the liberation of more deeply embedded compounds [12].

### 3.3. Effects of Ultrasonic Conditions on Antioxidant Capacity of E. tirucalli

As the antioxidant activities of a sample could vary depending on the efficiency of antioxidant assays used [16], the impact of UAE conditions on antioxidant capacity of *E.* *tirucalli* was determined using three antioxidant assays; ABTS, DPPH and CUPRAC. Analysis of the ABTS assays of the extracts revealed that antioxidant capacity was influenced by all three ultrasound bath variables, however, only ultrasonic temperature significantly affected antioxidant capacity (*p* < 0.05; Table 3). These data indicated that ultrasonic temperature was the major factor influencing ABTS antioxidant capacity, whereas, ultrasonic time and power had least impact on ABTS antioxidant capacity.

**Table 3:** Analysis of variance for the experimental results.

Parameter	DF	TPC		Antioxidant Capacity					
				ABTS		DPPH		CUPRAC	
		*F*	*p* > *F*	*F*	*p* > *F*	*F*	*p* > *F*	*F*	*p* > *F*
*β*_0_	1	16.90	<0.01 *	16.85	<0.01 *	8.91	<0.01 *	16.34	<0.01 *
*β*_1_	1	0.45	0.53	7.16	0.04 *	23.92	0.00 *	12.63	0.02 *
*β*_2_	1	1.78	0.24	0.62	0.47	1.46	0.28	0.32	0.59
*β*_3_	1	6.47	0.05 *	1.78	0.24	2.22	0.20	2.98	0.15
*β*_12_	1	7.24	0.04 *	0.01	0.92	5.96	0.06	0.35	0.58
*β*_13_	1	0.02	0.89	1.07	0.35	0.35	0.58	0.00	0.99
*β*_23_	1	0.02	0.90	0.31	0.60	0.86	0.40	0.26	0.63
*β*_11_	1	6.65	0.05 *	7.59	0.04 *	0.85	0.40	7.26	0.04*
*β*_22_	1	0.11	0.75	0.00	0.96	2.17	0.20	1.89	0.23
*β*_33_	1	2.50	0.17	7.75	0.04 *	0.10	0.77	11.59	0.02 *

* Significantly different at *p* < 0.05; *β*_0_: Intercept; *β*_1_, *β*_2_, and *β*_3_: Linear regression coefficients for temperature, time and power; *β*_12_, *β*_13_, and *β*_23_: Regression coefficients for interaction between temperature × time, temperature × power and time × power; *β*_11_, *β*_22_, and *β*_33_: Quadratic regression coefficients for temperature × temperature, time × time and power × power.

**Figure 3:**
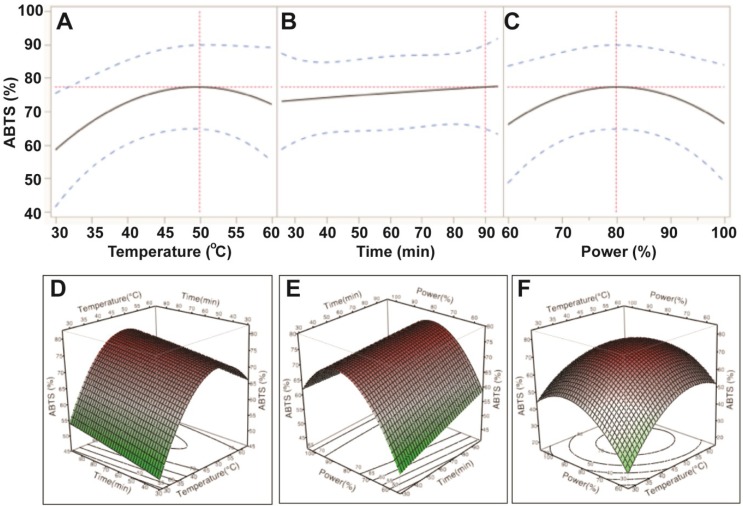
Impact of ultrasonic temperature (30–50 °C), time (30–90 min) and power (60%–100% or 150–250 W) on ABTS antioxidant capacity. The 2D impact of temperature, time and power were expressed in Figure 3A–C; while their 3D effects were shown in Figure 3D–F.

Table 3 indicates that the magnitude of the effect of the ultrasonic variables for ABTS antioxidant capacity was: temperature > power > time. ABTS antioxidant capacity increased when increasing the ultrasonic temperature from 30 °C to 50 °C and power from 150 W to 200 W. However, antioxidant capacity likely decreased when ultrasonic temperatures and ultrasonic power exceeded 50 °C and 200 W, respectively (Figure 3). Results from the ABTS assay revealed that the optimal conditions for maximum extraction of TPC could result in the highest antioxidant capacity. These findings can be explained by the high correlation between TPC and antioxidant capacity [15].

**Figure 4:**
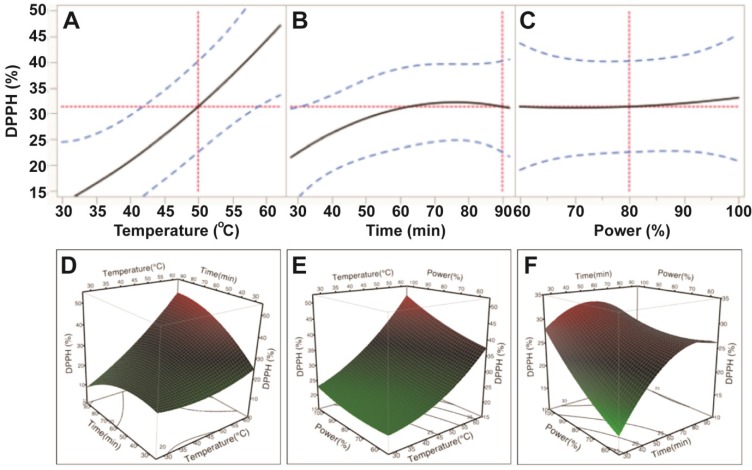
Impact of ultrasonic temperature (30–50 °C), time (30–90 min) and power (60%–100% or 150–250 W) on DPPH free radical scavenging capacity. The 2D impact of temperature, time and power were expressed in Figure 4A–C; while their 3D effects were shown in Figure 4D–F.

Data from the DPPH assay also indicated that free radical scavenging capacity of *E.* *tirucalli* extracts varied with the application of different ultrasonic temperature, time and power conditions; however, only temperature was reported to significantly affect free radical scavenging capacity (*p* < 0.05; Table 3). Based on the *p* values, the order of the influence was also found to be similar to ABTS: temperature > power > time. Free radical scavenging capacity of *E.* *tirucalli* extract increased steadily when ultrasonic temperature increased from 30 °C to 60 °C; whereas, its free radical scavenging capacity increased slightly with increasing power (150–250 W) and time (30–80 min; Figure 4). These data revealed that the highest DPPH free radical scavenging capacity of the extract was obtained at extraction conditions of 60 °C, 80 min and 250 W. At the optimal conditions for TPC (50 °C, 90 min and 200 W), only 66.5% of the maximum DPPH antioxidant value could be obtained.

**Figure 5:**
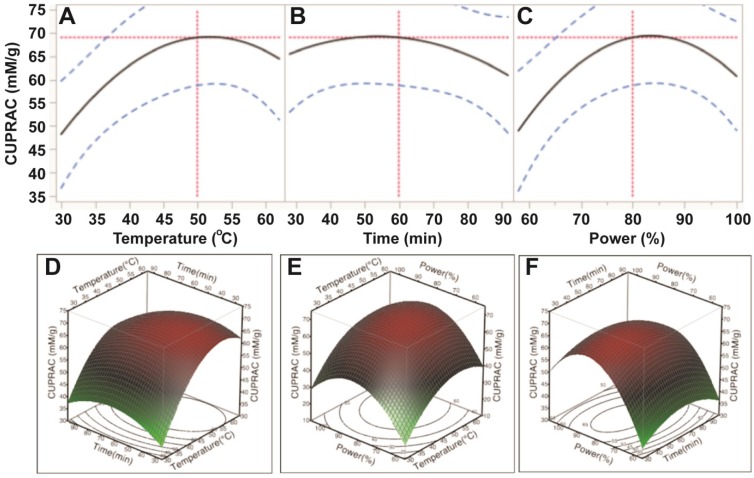
Impact of ultrasonic temperature (30–50 °C), time (30–90 min) and power (60%–100% or 150–250 W) on cupric reducing antioxidant capacity. The 2D impact of temperature, time and power were expressed in Figure 5A–C; while their 3D effects were shown in Figure 5D–F.

The results from the CUPRAC assay further confirmed that ultrasonic temperature had a significant impact on cupric reducing antioxidant capacity (CUPRAC) of the antioxidant capacity (*p* < 0.05). Ultrasonic time and power affected, but not significantly, the CUPRAC of the *E.* *tirucalli* extract (*p* > 0.05; Table 3). Based on the *p* values, the order of the effects was similar to those of ABTS and DPPH assays (temperature > power > time; Table 3). CUPRAC was observed to increase when increasing temperature from 30 °C to 50 °C, time from 30 min to 60 min and power from 150 W to 200 W; however, when ultrasonic temperature, time and power exceeded 50, 60 and 200 W, respectively, the CUPRAC of *E.* *tirucalli* extract decreased (Figure 5). These findings further confirmed that optimal extraction conditions for TPC could obtain maximum cupric reducing antioxidant capacity from *E.* *tirucalli* leaf.

The current findings showed that ultrasonic temperature, time and power affected the antioxidant capacity of the *E.* *tirucalli* extract; however temperature had the highest impact, followed by power, while time had the least impact on antioxidant capacity of the *E.* *tirucalli* extract. Sahin *et al*. [12] also found that ultrasonic temperature and time affected antioxidant capacity of an *Artemisia absinthium* extract and generally, antioxidant capacity increased when the time increased, but decreased when temperature exceeded 50 °C. Teh *et al*. [10] also reported that ultrasonic temperature and time influenced antioxidant capacity of extract from defatted hemp, flax and canola seed cakes. The impact of ultrasonic conditions on antioxidant capacity can be explained by the influence on the total phenolic compounds, which were found to contribute significantly to the antioxidant capacity of plant extracts [19,20,21].

### 3.4. Optimisation of Ultrasonic Extraction Conditions for Total Phenolic Content and Antioxidant Capacity of E. tirucalli

Previous epidemiological studies have established links between the consumption of foods containing high concentrations of phenolic and antioxidant compounds and a lower incidence of cardiovascular diseases and certain types of cancer [22,23,24]. An understanding of the factors affecting the extraction efficiency of phenolics and/or antioxidant compounds from plant sources is therefore important.

Based on the predictive models and values shown in Figure 2, Figure 3, Figure 4 and Figure 5, the optimum UAE conditions for the extraction of total phenolics, ABTS and CUPRAC from *E.* *tirucalli* leaf were determined to be: temperature = 50 °C, time = 90 min and power = 200 W; whereas, the optimum conditions for DPPH free radical scavenging capacity were: temperature = 60 °C, time = 80 min and power = 250 W. Of note, extraction under optimum conditions for TPC, ABTS and CUPRAC could obtain 66.5% of the maximum DPPH antioxidant value under its optimum conditions (60 °C, 80 min and 250 W). Therefore, the conditions of 50 °C, 90 min and 200 W were selected as optimal conditions for extraction of TPC and enhanced antioxidant capacity.

To validate the optimum conditions predicted by the models, *E.* *tirucalli* leaf was extracted under UAE conditions of 50 °C, 90 min and 200 W and the results showed that the predicted values of TPC and three antioxidant assays were similar to those of the experimental values (*p* > 0.05; Table 4). Therefore, these conditions were suggested for use to extract TPC and antioxidants from *E.* *tirucalli* leaf for further isolation and utilisation. In addition, these findings further confirmed the appropriateness of the models used for optimising the extraction conditions using UAE, and also revealed that response surface methodology was an effective technique for designing and optimising the extraction conditions.

**Table 4:** Validation of the predicted values for total phenolic content (TPC) and antioxidant capacity.

Variables	Values of TPC and Antioxidant Capacity	
	Predicted	Experimental (*n* = 4)
TPC (mg GAE/g FW)	3.32 ± 0.74 ^a^	2.93 ± 0.14 ^a^
ABTS (%)	77.26 ± 17.73 ^a^	71.50 ± 1.06 ^a^
DPPH (%)	31.33 ± 12.47 ^a^	35.24 ± 0.66 ^a^
CUPRAC (mM TAE/g FW)	61.77 ± 16.53 ^a^	54.03 ± 3.53 ^a^

All the values are means ± standard deviations and those in the same row not sharing the same superscript letter are significantly different from each other (*p* < 0.05).

## 4. Conclusions

As hypothesised, ultrasonic temperature, time and power had effects on extraction efficiency of total phenolic content and antioxidant capacity of the extracts from *E.* *tirucalli* leaf; however, the effects varied. Ultrasonic power had the highest impact on TPC; whereas, temperature had the strongest influence on antioxidant capacity. Levels of TPC and antioxidant capacity increased when ultrasonic temperature, time and power increased to 50 °C, 90 min and 200 W, respectively. Therefore, the optimum ultrasonic-assisted extraction conditions for TPC and antioxidant capacity from *E.* *tirucalli* leaf were: temperature of 50 °C, time of 90 min and power of 200 W. These conditions can be applied for further isolation and purification of phenolic compounds from the *E.* *tirucalli* leaf.
